# Towards a Central-Eastern European EQ-5D-3L population norm: comparing data from Hungarian, Polish and Slovenian population studies

**DOI:** 10.1007/s10198-019-01071-0

**Published:** 2019-05-17

**Authors:** Zsombor Zrubka, Dominik Golicki, Valentina Prevolnik-Rupel, Petra Baji, Fanni Rencz, Valentin Brodszky, László Gulácsi, Márta Péntek

**Affiliations:** 1 Department of Health Economics, Corvinus University of Budapest, Fővám tér 8, Budapest, 1093 Hungary; 2 Doctoral School of Business and Management, Corvinus University of Budapest, Fővám tér 8, Budapest, 1093 Hungary; 3 Department of Clinical and Experimental Pharmacology, Medical University of Warsaw, Banacha 1B, Warsaw, 02-097 Poland; 4 Institute for Economic Research, Kardeljeva ploščad 17, SI-1109 Ljubljana, Slovenia; 5 Premium Postdoctoral Research Program, Hungarian Academy of Sciences, Nádor u. 7, Budapest, 1051 Hungary

**Keywords:** EQ-5D-3L, Population norm, Central-Eastern Europe, Hungary, Poland, Slovenia, I10

## Abstract

**Background:**

EQ-5D-3L population data are available only from Hungary, Poland and Slovenia in Central and Eastern Europe (CEE). We aimed to compare the accessible studies and estimate a regional EQ-5D-3L population norm for CEE.

**Methods:**

A combined dataset using patient-level data of 8850 respondents was created. Based on the European Census of 2011, regional population norm estimates were calibrated by gender, age and education for the joint citizenry of 11 CEE countries.

**Results:**

EQ-5D-3L health states were available for 6926 and EQ VAS scores for 6569 respondents. Demographic characteristics of the samples reflected the recruitment methods (Hungary: online; Slovenia: postal survey, Poland: personal interviews). Occurrence of problems differed significantly by educational level in all the five dimensions (*p* < 0.001). The inter-country differences persisted after controlling for demographic variables. The estimated EQ-5D-3L index CEE norms with UK tariffs for age groups 18–24, 25–34, 35–44, 45–54, 55–64, 65–74 and 75 + were 0.911, 0.912, 0.871, 0.817, 0.762, 0.743 and 0.636 for males and 0.908, 0.888, 0.867, 0.788, 0.752, 0.68 and 0.584 for females, respectively. Estimates were provided also using Polish, European and Slovenian value sets.

**Conclusions:**

Besides gender and age, education should be considered during the design and interpretation of quality-of-life studies in CEE. The estimated regional EQ-5D-3L population norm may be used as a benchmark by CEE countries with lack of local dataset. However, the substantial inter-country differences in health status and scarcity of data over age 65 call for harmonized country-specific EQ-5D-3L population norm studies in the CEE region.

**Electronic supplementary material:**

The online version of this article (10.1007/s10198-019-01071-0) contains supplementary material, which is available to authorized users.

## Introduction

The EQ-5D-3L questionnaire (EQ-5D) is a generic health status measure that represents social preferences (utility scores) for health states; hence, it plays a key role in health economic evaluations [1]. Over the past two decades, formal health technology assessment (HTA) has been established in most Central and Eastern European (CEE) countries [2], and the majority of HTA guidelines in the CEE region prefer the EQ-5D to estimate quality-adjusted life-years (QALYs) for cost-effectiveness analyses. According to a recent review, between 2000 and 2015, 143 EQ-5D studies were conducted in a wide range of clinical areas in eight CEE countries [3]. However, EQ-5D normative data, representative for the entire population of a country, are available only for Hungary, Poland and Slovenia [4]. Although an increase in the number of EQ-5D publications from the CEE region has been observed since 2015, the scarcity of EQ-5D population norms is a major concern from both public health and HTA perspective. Population norms enable the comparison of the citizens’ health status both within a society and between countries. Comparing specific patient groups to their counterparts from the general public with similar socio-demographic characteristics allows the assessment of disease burden and enables decision makers to prioritize disease areas [4]. In the absence of a country-specific EQ-5D population norm, it is likely that data from other countries are used. However, such transfers require a good understanding of the drivers of inter-country differences. The application of an arbitrary dataset without adjustments might lead to biases and finally to inadequate health policy decisions.

Our aim was to compare three EQ-5D-3L studies conducted on large representative population samples in Central-Eastern Europe [3]. The second goal was to develop an estimated population norm for the CEE region based on accessible data and highlight the lessons to learn for future country-specific EQ-5D-3L studies in the region.

## Methods

### The EQ-5D-3L questionnaire

EQ-5D-3L is a generic quality-of-life instrument available in more than 170 languages worldwide [1]. The questionnaire consists of two parts. The descriptive system assesses self-reported health in five dimensions: mobility, self-care, usual activities, pain/discomfort and anxiety/depression. In each dimension, respondents are asked to describe their current health with one of the following three categories: no problems, some problems and severe problems. The descriptive system defines 243 (3^5^) distinct health states. The EQ-5D-3L index scores (utility values) attached to each health state are measured in valuation studies and reflect societal preferences. The EQ-5D-3L index score of one represents perfect health, zero represents death, and negative values represent “worse than dead” health states [5, 6]. The second part of the instrument is a 20-cm vertical visual analogue scale (EQ VAS) ranging from 0 (worst imaginable health) to 100 (best imaginable health).

### Data sources

We created a combined patient-level dataset from the three most recent EQ-5D-3L studies conducted on large representative population samples in CEE countries [3]. We received fully anonymous datasets for our study. The Hungarian data collection was part of the EuroVaQ project involving 10 countries with the aim to determine the monetary value of a QALY. Participants were randomly allocated into two samples: in one respondents evaluated their current health on the EQ VAS scale, while in the other on the EQ-5D descriptive system [7, 8]. The Polish data were provided from a population norm study, in which several quality-of-life instruments were used alongside the EQ-5D-3L [9]. The Slovenian data were collected in the Slovenian EQ-5D-3L valuation study [10]. Unlike the Hungarian survey, in the Polish and Slovenian studies both the descriptive system and the EQ VAS were administered to each respondent. The entire dataset was partitioned into an “EQ-5D-3L sample” for the analysis of self-reported health problems (descriptive system) and EQ-5D-3L index scores, and an “EQ VAS sample” for the analysis of EQ VAS scores. Key characteristics of the studies are summarized in Table 1.

**Table 1:** Summary of included studies

Country	Author, date	Year of data collection	Method of data collection	EQ-5D-3L version used	Sample contacted	Response	Fully completed^a^	Sampling	Highest educational level categories used
Hungary	Donaldson, 2010 [8] Baji, 2015 [19]	2009–2010	Web panel	Electronic	NA	starters: 11,178 (~ 10% start rate estimated)	EQ-5D-3L: 2281 (62.4% of eligible subjects) EQ VAS: 1924 (51.8% of eligible subjects)	Web panel representative for the adult population by age, gender and socio-economic status.	Low: Primary Middle: Vocational, secondary High: College, university
Poland	Golicki, 2015 [9]	2014	Personal interview	Paper based	10,562	3978 (36,6%)	3937 (98.9%)	Multiple-stage random sampling of the adult population from 65 geographic strata, based on region, type and size of localities.	Low: Primary not completed, primary Middle: High school, vocational school, secondary school (college without finals, college with finals, technical college without finals and technical college with finals). High: Post-secondary school, university (BsC), university (MsC, MA, MD), university (PhD or higher)
Slovenia	Prevolnik Rupel, 2001 [10]	2000	Postal survey	Paper based	3000	770 (25.6%)	708 (92.1%)	Representative sample of the general adult population.	Low: Not higher than primary education Middle: Higher than primary education, no university education High: University education

### Study sample

Respondents were included in the joint database if data on age, gender, educational attainment, EQ-5D-3L descriptive system and EQ VAS were fully available. For Hungary, either all EQ VAS or full EQ-5D-3L descriptive system results had to be available in the respective samples. The highest educational level was surveyed differently in the three studies (Table 1). Therefore, educational attainment was categorized as “low”, if respondents had not started secondary education, “middle” if respondents had only secondary education and “high” if the respondent had any education above secondary school. Due to the binary questions referring to education, only university education represented the high education group in the Slovenian sample. The same age groups were set for the analyses that are normally used in EQ-5D population norm studies: 18–24, 25–34, 35–44, 45–54, 55–64, 65–74 and 75 + years [4]. Moderate and severe problems in the EQ-5D-3L descriptive system were combined into a single “any problems” category.

### Calibration of data

We used the Eurostat 2011 European Census database to calibrate the joint population norm to homogenous general population data [11]. From the census, “no formal education” and International Standard Classification of Education (ISCED) level 1 (primary education) were categorized as “low”, ISCED level 2 and 3 (lower and upper secondary education) as “middle” and ISCED levels 4–6 (post-secondary, first and second stage tertiary education) as “high” education.

### EQ-5D-3L value sets used

A systematic review of EQ-5D studies between 2000 and 2015 indicated the use of several value sets in the CEE region [3]. The UK value set based on the time-trade-off method (TTO) [12] was used most frequently, followed by the Polish TTO-based tariffs [13], the VAS-based European [14] and Slovenian value sets [10]. We present the EQ-5D-3L index scores using all the four value sets.

### Statistical analysis

Statistical analyses were conducted using Stata 14.2 software package [15]. Descriptive statistics were used to summarize demographic data and health problems. The samples were compared to the general population using the $\chi^{2}$ test for independence. To inform the calibration strategy, in each dimension, the effects of age, gender, education and country on reported problems were analysed via linear probability models for intuitive interpretation of results. The association of EQ VAS and EQ-5D-3L index scores with the same socio-demographic variables was analysed via linear ordinary-least-squares regression (OLS). Pairwise differences between countries were assessed using *t* tests and post hoc Wald tests. The Breusch-Pagan test was performed to test heteroscedasticity. To account for heteroscedasticity, all models were calculated with robust standard errors.

For calculating the combined population norm, the dataset was divided into post-strata by age, gender, education and country. The following post-stratification calibration weights [16] were tested:

- “Equal’’: equal weighting between the three countries, assuming that the entire CEE population within each calibrated stratum is similar to the average of Hungary, Poland and Slovenia;
- “Population”: weighting that reflects the relative size of the general population of the three countries within each stratum, assuming that the entire CEE population within each calibrated stratum is similar to the combined population of Hungary, Poland and Slovenia;
- “Sample”: weighting reflects the number of available respondents within the sample in each stratum, assuming that the entire CEE population is equally similar to Hungary, Poland and Slovenia, with no differences between countries within any strata.

In post-strata with missing sample data from a country, weights reflected only the countries with available data. The combined population norm was calibrated to the average population of 11 CEE countries that had available data from the 2011 EU Population Census: Bulgaria, Croatia, Czech Republic, Estonia, Latvia, Lithuania, Hungary, Poland, Romania, Slovakia and Slovenia [11]. Census populations with missing or undetermined education (1.8%) were allocated proportionally to the low, middle and high categories in each age group. Standard errors were calculated using the linearized variance estimation method [17]. Confidence intervals were calculated using *t*-distribution with *N*-1 degrees of freedom, where *N* represents the entire sample size.

## Results

From the four datasets, 8850 complete responders were included in the analysis (Table 2). Altogether 770 respondents were included in the original Slovenian database; however, 62 cases (8.1%) were excluded due to missing data. The percentage of incomplete questionnaires was 4.7% below 75 and 18.8% above 75 years of age (*p* < 0.001). There were no incomplete responses in the Hungarian samples, and only four cases due to missing EQ VAS scores were excluded from the Polish sample.

**Table 2:** Summary of demographic characteristics of the samples and the general adult populations

	Sample						General Adult Population^a^			
	Hungary, EQ VAS sub-sample	Hungary, EQ-5D-3L sub-sample	Poland	Slovenia	EQ VAS Total	EQ-5D-3L Total	Hungary	Poland	Slovenia	CEE^b^
Total (N)	1924	2281	3937	708	6569	6926	8,142,665	30,896,617	1699,493	84 943 052
Gender (%)										
Male	50.5%	37.7%	46.8%	43.9%	47.6%	43.5%	46.6%	47.7%	49.1%	47.6%
Female	49.5%	62.3%	53.2%	56.1%	52.4%	56.5%	53.4%	52.3%	50.9%	52.4%
Education by age groups (%)										
18-24 years	11.4%	13.0%	11.6%	16.2%	12.0%	12.5%	10.6%	12.1%	10.0%	11.6%
Low	0.0%	3.0%	2.2%	3.5%	1.8%	2.6%	0.5%	3.3%	1.1%	3.3%
Middle	44.1%	36.7%	89.9%	86.1%	76.6%	71.2%	78.7%	84.0%	95.0%	84.0%
High	55.9%	60.3%	7.9%	10.4%	21.6%	26.2%	20.8%	12.7%	3.9%	12.7%
25-34 years	27.9%	24.8%	15.6%	19.2%	19.6%	19.0%	16.9%	19.7%	17.9%	18.4%
Low	0.0%	0.9%	2.6%	8.1%	2.1%	2.4%	0.7%	6.1%	0.6%	4.3%
Middle	49.3%	36.6%	50.9%	58.8%	51.1%	45.6%	60.1%	52.4%	70.2%	58.7%
High	50.7%	62.5%	46.5%	33.1%	46.8%	52.0%	39.2%	41.5%	29.2%	37.0%
35-44 years	17.8%	21.8%	16.4%	20.6%	17.3%	18.6%	18.8%	16.4%	17.7%	17.8%
Low	0.0%	2.2%	7.0%	9.6%	5.2%	5.4%	0.7%	8.7%	1.2%	4.2%
Middle	61.4%	52.6%	57.0%	64.4%	59.3%	56.2%	72.6%	64.1%	73.0%	69.5%
High	38.6%	45.2%	36.0%	26.0%	35.5%	38.4%	26.7%	27.2%	25.8%	26.3%
45-54 years	21.7%	22.3%	15.5%	16.1%	17.4%	17.8%	15.5%	17.3%	18.3%	16.5%
Low	0.0%	2.0%	8.0%	10.5%	5.3%	5.8%	1.2%	11.5%	3.1%	5.7%
Middle	57.8%	53.2%	69.0%	64.0%	64.4%	62.0%	78.2%	69.3%	78.0%	72.4%
High	42.2%	44.8%	23.0%	25.4%	30.2%	32.2%	20.5%	19.1%	18.9%	21.9%
55-64 years	14.3%	14.8%	20.1%	13.0%	17.7%	17.6%	17.6%	17.2%	16.2%	16.8%
Low	0.0%	3.0%	15.1%	16.3%	11.6%	11.9%	1.1%	21.4%	5.1%	10.3%
Middle	44.2%	37.1%	66.2%	60.9%	60.6%	57.8%	82.8%	62.5%	79.9%	70.8%
High	55.8%	59.9%	18.7%	22.8%	27.8%	30.4%	16.0%	16.1%	15.0%	18.9%
65-74 years	6.0%	3.0%	13.2%	10.0%	10.8%	9.5%	11.6%	9.0%	10.7%	10.4%
Low	0.0%	0.0%	31.9%	31.0%	26.6%	28.5%	2.1%	42.3%	9.8%	22.2%
Middle	32.2%	38.2%	53.7%	52.1%	50.1%	52.0%	83.6%	43.8%	78.1%	61.7%
High	67.8%	61.8%	14.4%	16.9%	23.3%	19.5%	14.4%	14.0%	12.1%	16.1%
75 + years	0.9%	0.3%	7.5%	4.8%	5.3%	4.9%	9.0%	8.1%	9.3%	8.6%
Low	0.0%	0.0%	46.5%	58.8%	45.3%	46.9%	5.6%	61.9%	18.2%	37.0%
Middle	33.3%	33.3%	39.1%	41.2%	39.0%	39.2%	84.9%	29.5%	73.6%	52.7%
High	66.7%	66.7%	14.5%	0.0%	15.8%	13.9%	9.5%	8.6%	8.1%	10.3%

^a^2011 Eurostat Census

^b^Bulgaria, Croatia, Czech Republic, Estonia, Latvia, Lithuania, Hungary, Poland, Romania, Slovakia, Slovenia

### Demographics

The mean age (± SD) was 45.4 ± 16.8 and 45.9 ± 17.1 years in the EQ-5D-3L and the EQ VAS samples, respectively. The rate of respondents with low, middle and high education was, respectively, 9.9, 56.1 and 33.9% in the EQ-5D-3L sample and 9.8, 58.8 and 31.4% in the EQ VAS sample. Table 2 summarizes the demographic attributes of the samples by country, the joint samples, the characteristics of the general populations of Hungary, Poland and Slovenia, and the combined CEE population involving 11 countries.

In Hungary, the mean age (± SD) was 40.8 ± 13.3 years (median 40, range 18–84) and 41.8 ± 14.2 years (median 40, range 18–86) in the EQ-5D-3L and EQ VAS samples, respectively. The proportion of the 65–74 and 75 + age groups was considerably lower in both samples than in the general population. While high education was generally over-represented, the scarcity of respondents with low education in the sample reflected properly the structure of the Hungarian general population. The percentages of responders with low, middle and high education were 2.0, 43.9 and 54.1% in the EQ-5D-3L sample, 0.0, 50.8 and 49.2% in the EQ VAS sample and 1.4, 76.2 and 22.3% the general population, respectively. The proportion of highly educated respondents increased with age. In the 65 + age group, 62.2% and 67.7% of Hungarian respondents had high education in the EQ-5D-3L (*n* = 74) and EQ VAS (*n* = 133) samples, respectively. This was in contrast with the statistics of the general population in which the peak of highly educated individuals was actually in age group 25–34 years. An over-representation of highly educated people can be observed in all age groups in the Hungarian samples, presumably due to internet-based recruitment and data collection.

In the Slovenian sample, the mean age (± SD) was 43.6 ± 17.6 years (median 41, range 18–92 years). The proportion of over 75-year-olds was slightly lower in the sample than in the general population. Moreover, low education was over-represented in all age groups. The percentages of low, middle and high education were, respectively, 13.8, 64.0 and 22.2% in the sample and 4.5, 77.3 and 18.9% in the general population. Contrary to the Hungarian sample, the percentage of low education increased and high education decreased with age, in line with the national population statistics. In the 65 + age group (*n* = 105) 40.0, 48.6 and 11.4% had low, middle and high education, respectively.

The Polish sample was similar to the general population in most demographic parameters. Mean age (± SD) was 48.3 ± 17.8 years (median 49, range 18–87). The percentages of low, middle and high education were, respectively, 13.8, 61.8 and 24.4% in the study sample and 17.4, 60.2 and 22.3% in the general population. As in the Slovenian sample, the percentage of low education increased and high education decreased with age. In the 65 + age group (*n* = 818), 37.2, 48.4 and 14.4% of respondents had low, middle and high education, respectively.

### Health problems

**Fig. 1:**
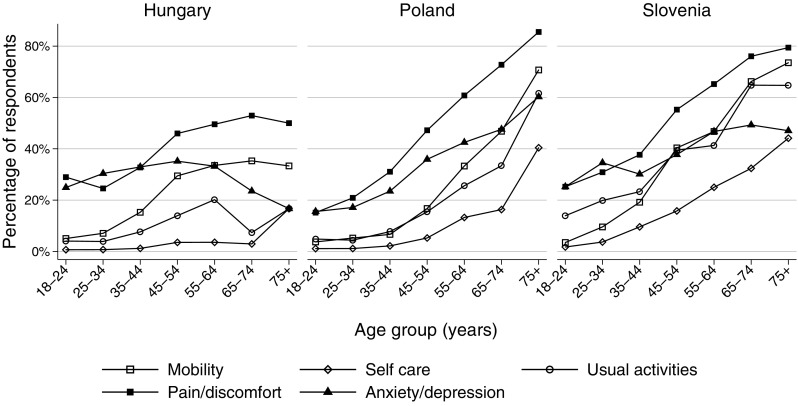
Any problems reported by EQ-5D-3L dimensions. In the Hungarian sample, there were 74 respondents aged 65 + and 100% of them reported any problem

The percentages of respondents reporting any problems in each EQ-5D-3L dimension are shown by countries in Fig. 1. In the entire sample, pain/discomfort problems occurred most frequently (42.7%), followed by anxiety/depression (33.0%). Problems with self-care were least frequent (7.4%). Problems with mobility and usual activities occurred in 17.3% and 22.2% of respondents, respectively. In all the five dimensions problems were reported most frequently by Slovenian, followed by Polish and Hungarian respondents. Any problems with mobility were reported by 29.1, 23.2 and 18.4% (*p* < 0.001), with self-care by 14.1, 9.4 and 2.0%, (*p* < 0.001), with usual activities by 32.2, 19.1 and 9.5% (*p* < 0.001), with pain/discomfort by 46.6, 45.8 and 36.3% (*p* < 0.001), and by anxiety/depression by 36.3, 33.3 and 31.5% (*p* = 0.049) by Slovenian, Polish and Hungarian respondents, respectively.

Education influenced significantly the occurrence of health problems in all dimensions of the EQ-5D-3L. In the entire sample, problems with mobility were reported by 50.7, 21.7 and 14.6% (*p* < 0.001), with self-care by 27.2, 6.7 and 2.7% (*p* < 0.001), with usual activities by 45.4, 17.6 and 8.6% (*p* < 0.001), with pain/discomfort by 71.3, 44.1 and 32.0% (*p* < 0.001) and with anxiety/depression by 53.6, 33.5 and 26.2% (*p* < 0.001) by respondents with low, middle and high education, respectively.

In the 65 + age group, the occurrence of problems was lower in the Hungarian sample (*n* = 74) compared to Poland (*n* = 818) and Slovenia (*n* = 105) in all dimensions. In this age group, any problems with mobility were reported by 35.1, 55.5 and 68.6% (*p* < 0.001), with self-care by 4.1, 25.1 and 36.2% (*p* < 0.001), with usual activities by 8.1, 43.6 and 64.8% (*p* < 0.001), with pain-discomfort by 52.7, 77.4 and 77.1% (*p* < 0.001) and with anxiety-depression by 23.0, 52.2 and 48.6% (*p* < 0.001) by Hungarian, Polish and Slovenian respondents, respectively. Further details about the occurrence of problems by age, gender and education are provided in the Online Resource (Supplementary Table S1).

### EQ VAS scores

**Fig. 2:**
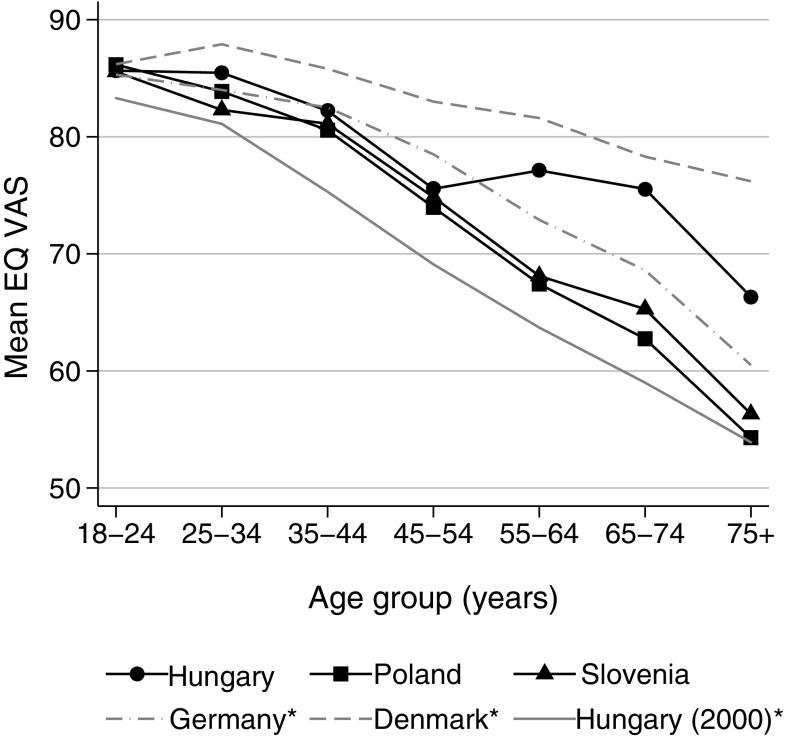
Comparison of country EQ VAS scores by age groups. *Source: Szende et al. (2014) [4]. Mean (SE) EQ VAS scores in the 65 + age group: Hungary 74.3 (1.6); Poland 59.7 (0.7); Slovenia 62.4 (1.9)

The average EQ VAS scores in comparison to European countries with the highest (Denmark, 2000-2001), lowest (another previous study from Hungary, 2000) and middle (Germany, 2001–2002) population EQ VAS scores [4] are depicted in Fig. 2. Up to 55 years of age, the average EQ VAS scores of Hungary, Poland and Slovenia were similar, in the lower range of the European values. However, the scores of the Hungarian sample were higher by 9.7 (*p* < 0.001), 12.8 (*p* < 0.001), 12.0 (*p* < 0.001) points than the Polish, and by 9.0 (*p* < 0.001), 10.3 (*p* < 0.001) and 10.0 (*p* = 0.04) points than the Slovenian scores in the 55–64, 65–74 and 75 + age groups, respectively.

### EQ-5D-3L index score comparisons using different value sets

The index score difference between respondents with low and high education was 0.214 (*p* < 0.001), 0.141 (*p* < 0.001), 0.201 (*p* < 0.001) and 0.248 (*p* < 0.001) using the UK, Polish, European and Slovenian value sets, respectively. The gender-related differences were significant, although more moderate. Men had higher index scores by 0.028 (*p* < 0.001), 0.0165 (*p* < 0.001), 0.029 (*p* < 0.001) and 0.034 (*p* < 0.001) using the UK, Polish, European and Slovenian value sets, respectively. The age-related differences were smallest in the Hungarian sample and greatest in the Polish one with all value sets. The difference between the 18–24 and 65 + age groups was 0.086, 0.054, 0.082 and 0.123 in the Hungarian sample, 0.225, 0.140, 0.232 and 0.335 in the Slovenian sample and 0.285, 0.183, 0.273 and 0.347 in the Polish sample, using the UK, Polish, European and Slovenian value sets, respectively. Using conservative p values after Bonferroni correction (*p* = 0.05/126 = 0.00039) due to multiple testing, most value set comparisons in most age groups were significantly different. Exceptions were the UK and European value sets, which were similar in several age groups of the Hungarian and Slovenian samples. After Bonferroni correction (*p* = 0.05/12 = 0.0042), Hungary was significantly different from both Poland and Slovenia in all value sets, while Poland and Slovenia did not differ significantly in either of them. The index scores by age groups calculated by four value sets for each country are shown in the Online Resource (Supplementary Figure S2).

### Country-specific differences after controlling for demographic variables

The aim of the regression analyses was to detect whether country-specific health status differences exist after controlling for age, gender and education level. Supplementary Table S3 of the Online Resource summarizes the results of any problems, EQ-5D-3L index- and EQ VAS scores. As we described above, data were obtained from two different samples for EQ-5D-3L and EQ VAS in Hungary. The Breusch–Pagan test indicated heteroscedasticity in all models. All models were statistically significant, albeit with low explanatory power. *R*^2^ was highest for the EQ VAS model (0.249) and lowest for any problems with anxiety/depression (0.072).

The main effects of education (*p* = 0.0375 to < 0.001), age (*p* = 0.0583 to < 0.001) and gender (*p* = 0.613 to < 0.001) were statistically significant across all health problems, all EQ-5D-3L index models and the EQ VAS model, with the exception of self-care problems, where the difference between men and women was not significant, and anxiety/depression, where the age-related differences were not significant.

The country─education interaction revealed significant country-specific differences in mobility (*p* = 0.0013), usual activities (*p* = 0.007) and pain/discomfort dimensions (*p* = 0.0011). The greatest difference of coefficients between the high and low education groups was found in the pain/discomfort dimension in Hungary (− 0.407, *p* < 0.001), and usual activities in Slovenia (− 0.402, *p* < 0.001). The education-related differences were less pronounced in Poland, with greatest difference in the usual activities dimension (− 0.170, *p* < 0.001). For the EQ-5D index scores, the main effect and country interactions of education were significant for all value sets (*p* < 0.001 for all). For the UK TTO index score model, differences of coefficients between high and low education status were 0.198 (*p* < 0.001) and 0.213 (*p* < 0.001) in Hungary and Slovenia, respectively. The education-related difference was smallest in Poland (0.094, *p* < 0.001).

The country─age group interactions were significant in all dimensions (*p* = 0.0018 to < 0.001). The greatest differences of coefficients between age groups were found in all countries in the pain/discomfort dimension, with highest differences in Poland (0.672, *p* < 0.001), lowest ones in Hungary (0.272, *p* < 0.001) and Slovenian ones (0.477, *p* < 0.001) in between. Country─age group interactions of index scores were significant for the Polish TTO, UK TTO and European VAS value sets (*p* < 0.000 for all). The UK TTO index score differences between the 18–24 and 65 + age groups were the greatest in Poland (0.260, *p* > 0.001), and the smallest in Hungary (0.102, *p* < 0.001), with Slovenia (0.171, *p* < 0.001) in the middle.

The country─gender interaction was significant only in the anxiety/depression dimension (*p* = 0.0243). Female gender increased most the coefficient of anxiety/depression (0.110, *p* < 0.001) in Hungary, and pain/discomfort in Slovenia (0.038, *p* < 0.001) and Poland (0.061, *p* < 0.001). In the UK TTO index score model, gender-related differences were 0.037 (*p* < 0.001), 0.021 (*p* < 0.01) in Hungary and Poland. The difference was minimal and statistically not significant in Slovenia (0.001, *p* > 0.05). Gender interaction was not significant in any of the EQ-5D-3L index models.

### Joint population norm for the CEE region

Our findings confirmed that due to the significant effect of education on EQ-5D-3L index scores, it was necessary to calibrate the sample for educational level in addition to gender and age groups. Due to the country-specific differences in the effect of age and education on the EQ-5D-3L index scores, we assumed that equal country weighting would provide most valid estimates for the CEE region. However, in 7 (5.5%) out of the 126 gender–age–education–country strata, data were missing. Also, in 60 strata (47.6%) the sample size was less than 30, which is considered desirable for post-stratum weighting [16]. To create strata with acceptable sample sizes and avoid extreme weights, we chose a “mixed” weighting strategy. In the middle education group, up to 64 years of age, and in the high education groups between 25 and 65 years of age gender─age─education─country strata were formed with “equal” country weighting. In low education groups, ages above 65 years and in the 18–24-year-old age group with high education, gender─age─education strata were formed with “sample” weighting between countries. Altogether the number of strata was reduced to 78, out of which only 14 (18%) had sample sizes slightly below 30. Weights ranged between 0.3 and 5.63. Due to the very low or missing respondent counts, some strata in the 65 + age group reflected information from one or two countries only, and the estimates were less precise than in younger age groups.

To test the mixed weighting results, we estimated the combined population norms for all value sets with “equal”, “population” and “sample” weighting. In vast majority of age groups, 95% confidence intervals overlapped for all weighting strategies using all value sets, suggesting no significant differences between the four strategies. (Regional population norms estimated with different weighting strategies using the UK TTO value set are illustrated in Supplementary Figure S4 of the Online Resource.) We concluded that the “mixed” weighting strategy may be appropriate to calculate a population norm that is representative of the CEE population. Our results are presented for the UK, Polish, European and Slovenian value sets in Table 3. The weighted relative problem frequencies by each EQ-5D-3L dimension are also provided in Table 4.

**Table 3:** Estimated EQ-5D-3L index population norm values for the CEE region

	Polish TTO value set		UK TTO value set		Slovenian VAS value set		European VAS value set	
	Male	Female	Male	Female	Male	Female	Male	Female
18–24 years	0.953	0.950	0.911	0.908	0.894	0.887	0.902	0.898
	[0.942–0.963]	[0.941–0.959]	[0.890–0.931]	[0.893–0.923]	[0.872–0.915]	[0.870–0.903]	[0.882–0.922]	[0.883–0.912]
25–34 years	0.950	0.940	0.912	0.888	0.888	0.861	0.9027	0.875
	[0.939–0.961]	[0.931–0.949]	[0.894–0.929]	[0.873–0.904]	[0.869–0.907]	[0.844–0.877]	[0.885–0.919]	[0.86–0.891]
35–44 years	0.924	0.927	0.871	0.867	0.836	0.830	0.863	0.853
	[0.907–0.942]	[0.919–0.935]	[0.847–0.894]	[0.854–0.881]	[0.811–0.861]	[0.813–0.847]	[0.842–0.883]	[0.839–0.867]
45–54 years	0.891	0.876	0.817	0.788	0.771	0.733	0.813	0.782
	[0.871–0.911]	[0.863–0.890]	[0.790–0.845]	[0.766–0.809]	[0.743–0.799]	[0.708–0.758]	[0.790–0.836]	[0.762–0.802]
55–64 years	0.858	0.855	0.762	0.752	0.686	0.683	0.752	0.743
	[0.840–0.876]	[0.839–0.871]	[0.737–0.787]	[0.728–0.777]	[0.656–0.716]	[0.655–0.71]	[0.729–0.775]	[0.721–0.765]
65–74 years	0.843	0.805	0.743	0.680	0.674	0.597	0.740	0.679
	[0.821–0.865]	[0.783–0.827]	[0.714–0.773]	[0.653–0.708]	[0.645–0.704]	[0.571–0.624]	[0.716–0.765]	[0.657–0.702]
75 + years	0.781	0.731	0.636	0.584	0.528	0.495	0.628	0.595
	[0.750–0.812]	[0.698–0.764]	[0.591–0.680]	[0.545–0.623]	[0.485–0.572]	[0.461–0.528]	[0.589–0.666]	[0.566–0.625]

Weighted mean estimates using “mixed” weighting strategy [95% CI]

**Table 4:** Estimated relative problem frequencies by EQ-5D-3L dimensions

Sample N	Age group (years)						
	18–24	25–34	35–44	45–54	55–64	65–74	75+
	491	751	754	686	642	364	224
Males							
Mobility							
Some problems	3.7%	7.1%	14.3%	25.9%	39.0%	38.8%	65.8%
	[1.9%–7.1%]	[4.7%–10.5%]	[10.5%–19.2%]	[21.3%–31.2%]	[33.3%–45.0%]	[33.4%–44.5%]	[56.6%–73.9%]
Confined to bed	0.0%	0.1%	1.1%	0.1%	1.0%	2.5%	0.7%
	[0.0%–0.0%]	[0.0%–0.6%]	[0.3%–4.1%]	[0.0%–0.8%]	[0.3%–3.2%]	[1.2%–5.1%]	[0.1%–4.7%]
Self-care							
Some problems	0.7%	1.5%	3.5%	7.2%	14.8%	12.4%	34.5%
	[0.2%–2.1%]	[0.6%–3.7%]	[1.8%–7.0%]	[4.5%–11.3%]	[10.8%–20.0%]	[9.1%–16.7%]	[26.4%–43.6%]
Unable to	0.0%	0.0%	1.1%	0.1%	0.6%	1.3%	4.0%
	[0.0%–0.0%]	[0.0%–0.0%]	[0.3%–4.1%]	[0.0%–0.8%]	[0.2%–1.5%]	[0.5%–3.5%]	[1.5%–10.2%]
Usual activities							
Some problems	8.5%	9.7%	13.4%	19.0%	27.8%	27.4%	51.3%
	[5.3%–13.4%]	[6.7%–13.8%]	[9.7%–18.3%]	[14.9%–24.0%]	[22.7%–33.6%]	[22.7%–32.7%]	[42.1%–60.3%]
Unable to	0.0%	0.3%	0.5%	0.3%	1.6%	2.5%	3.6%
	[0.0%–0.0%]	[0.0%–1.8%]	[0.2%-1.7%]	[0.1%-1.1%]	[0.9%-2.7%]	[1.2%-5.1%]	[1.3%-9.4%]
Pain/discomfort							
Some	23.0%	19.4%	30.0%	42.2%	57.8%	59.7%	78.5%
	[17.4%-29.7%]	[15.4%-24.1%]	[25.2%-35.4%]	[37.0%-47.6%]	[51.8%-63.6%]	[54.0%-65.1%]	[69.8%-85.2%]
Extreme	0.3%	1.1%	1.0%	3.4%	2.6%	4.0%	3.6%
	[0.0%–1.8%]	[0.3%–3.4%]	[0.3%–4.0%]	[1.7%–6.7%]	[1.5%–4.4%]	[2.2%–6.9%]	[1.3%–9.4%]
Anxiety/depression							
Some	19.2%	22.4%	23.9%	30.3%	38.0%	37.3%	55.8%
	[14.1%–25.6%]	[18.1%–27.4%]	[19.5%–29.0%]	[25.6%–35.5%]	[32.3%–44.1%]	[31.9%–42.9%]	[46.6%–64.6%]
Extreme	2.1%	0.6%	1.2%	3.1%	1.7%	1.7%	1.8%
	[0.7%–5.7%]	[0.2%–2.3%]	[0.5%–2.7%]	[1.5%–6.4%]	[0.8%–3.6%]	[0.7%–4.0%]	[0.4%–7.1%]
Females							
Mobility							
Sample N	491	751	754	686	642	364	224
Some problems	4.5%	7.1%	11.3%	29.0%	37.4%	52.0%	69.0%
	[2.8%–7.0%]	[5.2%–9.8%]	[9.0%–14.3%]	[24.5%–34.0%]	[31.9%–43.3%]	[46.7%–57.2%]	[62.3%–75.0%]
Confined to bed	0.0%	0.0%	0.1%	0.5%	0.1%	1.4%	2.0%
	[0.0%–0.0%]	[0.0%–0.0%]	[0.0%–0.5%]	[0.2%–1.2%]	[0.0%–0.5%]	[0.6%–3.5%]	[0.8%–4.9%]
Self-care							
Some problems	1.0%	2.1%	3.7%	7.5%	12.4%	17.1%	35.9%
	[0.4%–2.5%]	[1.0%–4.2%]	[2.1%–6.3%]	[4.9%–11.3%]	[8.7%–17.4%]	[13.6%–21.4%]	[29.8%–42.5%]
Unable to	0.0%	0.0%	0.1%	0.3%	0.1%	1.4%	2.7%
	[0.0%–0.0%]	[0.0%–0.0%]	[0.0%–0.5%]	[0.1%–1.1%]	[0.0%–0.5%]	[0.6%–3.5%]	[1.3%–5.7%]
Usual activities							
Some problems	6.3%	8.4%	10.2%	24.6%	28.2%	33.8%	56.7%
	[4.1%–9.6%]	[5.9%–11.8%]	[7.7%–13.3%]	[20.1%–29.6%]	[23.1%–33.8%]	[29.0%–38.9%]	[49.8%–63.3%]
Unable to	0.1%	0.5%	0.5%	0.9%	1.4%	3.6%	5.8%
	[0.0%–1.0%]	[0.1%–3.1%]	[0.2%–1.3%]	[0.5%–1.8%]	[0.4%–4.8%]	[2.1%–6.2%]	[3.5%–9.5%]
Pain/discomfort							
Some	25.2%	28.8%	35.8%	51.8%	58.4%	72.3%	73.8%
	[21.1%–30.0%]	[24.9%–33.0%]	[31.7%–40.1%]	[46.8%–56.8%]	[52.6%–64.0%]	[67.4%–76.8%]	[67.4%–79.3%]
Extreme	0.5%	0.6%	0.7%	2.5%	3.4%	5.1%	11.3%
	[0.1%–2.3%]	[0.1%–2.8%]	[0.3%–1.5%]	[1.6%–3.8%]	[2.1%–5.4%]	[3.2%–8.0%]	[7.8%–16.2%]
Anxiety/depression							
Some	21.4%	30.1%	30.5%	35.4%	43.4%	45.8%	55.3%
	[17.4%–26.0%]	[26.0%–34.7%]	[26.6%–34.7%]	[30.8%–40.3%]	[37.6%–49.3%]	[40.7%–51.1%]	[48.5%–62.0%]
Extreme	1.5%	1.0%	1.1%	3.5%	2.7%	4.1%	3.0%
	[0.6%–3.5%]	[0.5%–2.0%]	[0.6%–2.0%]	[2.1%–5.7%]	[1.2%–5.8%]	[2.4%–6.7%]	[1.4%–6.4%]

Weighted relative frequency estimates using “mixed” weighting strategy [95% CI]

## Discussion

Our study is an in-depth comparison of three EQ-5D-3L studies originating from Hungary, Poland and Slovenia and involving large representative samples of the general population. Our findings demonstrated that educational attainment has a major influence on the health status of the general populations of these countries. Despite their similar economic status and historical background, we found that the effect of age and educational level on the occurrence of health problems as well as on EQ-5D-3L index scores was different between the three countries. After calibration to the combined general population of 11 CEE countries in terms of gender, age and education, we estimated population norms for the CEE region, using the UK, Polish, European and Slovenian value sets.

The main strength of our research is that it was conducted on a joint database including data from 8850 individual respondents. EQ-5D-3L health states were available from 6926 and EQ VAS scores from 6569 respondents with corresponding age, gender and education status. To our knowledge, as of the writing of this article, only six published national EQ-5D-3L surveys involved larger EQ-5D-3L or EQ VAS samples than our joint database [4, 18].

First, we highlight two important points to consider for the interpretation of the results. One is the differences in recruitment and data collection methods between the three countries, which are reflected by the different sample profiles. The other is that there was a 14-year difference between the three studies (2000 in Slovenia, compared to 2014 in Poland). Before discussing our findings in detail, we provide some considerations regarding these two issues.

The study ran online in Hungary, while it was a face-to-face interview in Poland and postal survey in Slovenia. According to a recent review by Janssen and colleagues, among the EQ-5D population studies conducted in 20 countries between 1994 and 2010, 17 (85%) were personal interviews, 3 (15%) were postal surveys and none of them was conducted online [18]. Between 2010 and 2015 in eight CEE countries, 88% of EQ-5D studies were conducted on-site, while 4% were online studies and 4% were postal surveys [3].

In the 65 + age group of the Hungarian online sample, we found low proportions of complete responses. Elderly respondents in the Hungarian sample were predominantly highly educated, while the Polish face-to-face sample showed no systemic deviations from the general population. Interestingly, in the online EuroVaQ study response rates in the 65 + age group were similarly low in both Hungary and Poland. However, in the same study, the response rates of older and younger participants were similar in most Western-European countries [8]. The online selection bias may contribute to the notably higher average Hungarian EQ-5D-3L index scores compared to Poland or Slovenia in the 65 + age group. The average European EQ-5D-3L index score of Hungary was higher by 0.145 (*p* < 0.001) than that of Poland and by 0.153 (*p* < 0.001) than that of Slovenia. In addition to our online survey from 2010, in Hungary an EQ-5D-3L population survey was conducted via personal interviews in 2000 (*n* = 5503). The European VAS index scores of the online survey were also greater by, respectively, 0.09 and 0.14 in the 65–74 and 75 + age groups, compared to the published values of the face-to-face survey. (*P* values are not available) [19].

The Polish study was conducted via qualified interviewers and paper–pencil questionnaires, in order to resemble most the way quality of life questionnaires are administered in Poland [9]. However, online versions of EQ-5D instruments are widely available [5] and may gain popularity over time. Despite the cost and convenience benefits of online surveys, the potential for coverage error can be high [20], which may be especially important in the CEE region in older individuals with low education levels, who have seemingly limited willingness and/or ability to participate in online surveys. In the EuroVaQ study, despite the increased incentives, some demographic groups were difficult to reach online [8]. Therefore, in population health surveys, mixing online and offline methods could be considered to increase coverage in hard to reach populations [20, 21].

The time difference between the surveys (Slovenia 2000, Hungary 2010, Poland 2014) deserves attention because some key healthcare indicators have shown considerable changes over this time period in the three countries. In 2000, 2010 and 2014, healthcare spending as a % of GDP was 6.8, 7.6 and 7.1% in Hungary, 5.3, 6.4 and 6.2% in Poland and 7.8, 8.6 and 8.5% in Slovenia, respectively. In the same years, healthcare expenditure per capita in constant PPP 2010 US dollars increased by approximately 40% in Hungary ($1166, $1622 and $1652) and Slovenia ($1714, $2380 and $2356), whilst nearly doubled in Poland ($756, $1354 and $1474). Life expectancy at birth increased by 4–5 years during this period in all the three countries (in 2000, 2010 and 2014: 71.9, 74.7 and 75.9 years in Hungary; 73.8, 76.5 and 77.5 years in Poland; and 76.1, 79.8 and 81.2 years in Slovenia, respectively) [22]. Although the Slovenian study was conducted a decade earlier than the Hungarian and Polish ones, healthcare expenditure and life expectancy of Slovenia in 2000 were comparable to the 2010 and 2014 values of Hungary and Poland. While life expectancy in Hungary improved between 2000 and 2010, despite the chronic financial difficulties of the healthcare system [23], in-depth comparison of the Hungarian EQ-5D-3L population studies from 2000 to 2010 did not indicate significant change in the self-reported health status of the population [4, 18]. In the Hungarian study from 2000, problems were reported less frequently in all dimensions than in the Slovenian study from the same year [4]. However, the Hungarian EQ VAS values were lower in every age group compared to the Slovenian ones, which suggests that country-specific reporting differences might also exist. Also, despite the similar economic situation of CEE countries, unequal access to healthcare services has been described [24, 25]. Therefore, although the methodology and time of the surveys influenced the results, especially in the 65 + age group, we cannot conclude that the differences between the self-reported population health status of the three countries can be explained by methodological differences alone.

Our analyses brought into focus both important regional specificities and inter-country differences. Problems were reported more frequently in Slovenia than in Poland or Hungary and any other European country with available EQ-5D-3L population norms, with the exception of the pain/discomfort dimension [4, 18]. Poland would also rank among top three in the frequency of problems in all dimensions among European countries. The rate of self-reported problems in the anxiety/depression dimension deserves particular attention as it was the highest in Slovenia, Poland and Hungary among the European countries. It has been demonstrated that the frequency of problems in the anxiety/depression dimension shows strong negative correlation with the per capita GDP based on purchase-power parity [26]. The severe mental health problems of CEE countries are multifactorial, including high burden of substance and alcohol abuse, problems with the organization of healthcare services and inadequate resource allocation [27].

Our analyses revealed also that education had a major influence on the occurrence of health problems and EQ-5D-3L index scores, with significant differences between the three countries. Education is an important socio-economic determinant of health via both direct and indirect effects through occupation and income status [28]. The education-related health inequality varies from country to country [29]. In the CEE region, greater education-related health inequalities were found than in other European countries, especially in preventable causes of death [30]. Hungary and Poland were among top three countries in Europe with education-related differences of mortality in men and women, while the inequality was less pronounced in Slovenia [31]. In self-reported poor health, educational differences were reported most frequently in Hungary and Slovenia in the European Social Survey [32]. In EQ-5D population norm studies, Slovenia ranked highest and Hungary ranked second in the absolute health inequality explained by educational differences, and Hungary and Slovenia ranked in top five in the effect of education on health problems on all health dimensions [4]. When comparing socio-economic determinants of health, educational attainment is one of the most universally available and easy-to-compare variables [28]. In our analysis, the “high” education group consisted of any education above secondary school. In the Polish and Hungarian samples, there were post-secondary school or college categories below university education, while in Slovenia respondents could be categorized only to secondary or university education. In the 2011 Census data, 2.8% of the Polish and 4.1% of the Hungarian population had post-secondary education (ISCED 4) below tertiary level, while this category was empty in the Slovenian census data. Therefore, we concluded that the educational categories of the samples matched with that of the European Census were consistent in the three countries.

We provided population norm estimates calibrated to gender, age and educational status of the combined population of 11 CEE countries. In the 11 CEE countries 10.1, 67.6 and 22.3% of the population had low, middle and high education, respectively. However, there are differences in the educational status of the individual country populations within the CEE region, which deserve attention when interpreting our estimates. Low education has high prevalence in Poland (17.45%) and Romania (12.43%), while it is rather infrequent in Slovakia (0.13%), the Czech Republic (0.82%) and Hungary (1.4%). High education is more frequent in Lithuania (48.6%), Estonia (40.5%) and Latvia (38.3%) than in other countries in the region. Furthermore, the pattern of education-related health inequalities has changed over time in CEE countries [33], which further influences the precision of our estimates. Although our results underpin the importance of education when comparing the health status of populations and estimating the population norm for the CEE region, we have to note that our regression models had low explanatory power, with *R*^2^ lower than 0.243, suggesting that a major part of individual health variance was due to unobserved causes.

We observed country-specific differences between the effect of age and education on the EQ-5D-3L index scores in all the four (UK, Polish, European, Slovenian) value sets. To reflect these differences during calibration, ideal post-stratification weighting would require 42 gender–age–education strata by country, each with at least 30 respondents. To meet this condition, at least 541 additional respondents would be needed in the Hungarian sample (+ 24%), 161 in the Polish one (+ 4%) and 658 in the Slovenian one (+ 93%). Given the relatively low response rates, the costs of extensive data collection for covering all education groups may be substantial. Despite the sample size limitations, using any weighting strategy at any time point, the absolute difference between the weighted EQ-5D-3L index scores was not greater than 0.029, 0.023, 0.032 and 0.024 using the Polish, UK, Slovenian and European value sets, respectively, suggesting sufficiently accurate population norm estimates across all age groups, albeit with somewhat less precision in the 65 + age groups.

Population norm values by age groups were published in 2018 for 20 countries using the European VAS value set, without separately reporting data for males and females [18]. Due to the significant gender differences, we reported the CEE population norms separately for both sexes. The confidence intervals of estimated European VAS CEE population norms of males and females were significantly lower than the population norms of most Western-European countries, were closer to those of Hungary and were somewhat higher than those of Slovenia in most age groups. The international comparison suggests that our CEE population norm estimates provide a good solid benchmark for CEE countries without local population norms.

Some limitations of our study have to be noted. First, the differences between the three countries’ population health profiles can be attributed to different national characteristics, different data collection methodologies and different times of the three studies. The effects of these differences are not fully separable in our results. Also, the gaps in the available data prevented a fully consistent weighting of the regional EQ-5D-3L index population norm estimates for the 65 + age group, an extremely important population from public health and health economic point-of-view.

## Conclusions

Our in-depth analysis provides insights into the challenges of comparing and transferring the results of health surveys between countries. Our findings highlight the importance of taking education into account when collecting, reporting and interpreting quality-of-life data in the CEE region, where education-related health inequalities are more pronounced than in most European countries. The estimated regional EQ-5D-3L population norm may be used as a benchmark by CEE countries without local population norms. However, despite our attempt to provide as precise estimate as possible by calibrating our results by gender, age, education and country, our results have limitations, especially due to the low sample sizes in low education groups and the elderly. Also, a large share of individual health variance has not been accounted for by our analysis. Therefore, we strongly recommend performing sensitivity analysis when using the CEE population norm estimates for economic analysis.

We believe that our results contribute to the design of high-quality EQ-5D population norm studies in the CEE region. Due to the similar economic status and historical background, CEE countries may be viewed as a homogenous group and ideal candidates to transfer the results of health economic analyses between each other. However, despite the economic similarities, the differences of the health status and health utilities within the CEE region can be substantial. Therefore, further studies are needed to understand the key determinants of inter-country variability measured by the EQ-5D-3L and the more recent EQ-5D-5L instruments in various research settings, to make health economic analyses more comparable and transferable within the region.

## Electronic supplementary material

Below is the link to the electronic supplementary material.

Supplementary material 1 (PDF 140 kb)

Supplementary material 2 (PDF 94 kb)

Supplementary material 3 (PDF 93 kb)

Supplementary material 4 (PDF 241 kb)
