# Supplementary material for: Towards a Central-Eastern European EQ-5D-3L population norm: comparing data from Hungarian, Polish and Slovenian population studies
Source: Eur J Health Econ. 2019 May 17;20(Suppl 1):141–54. doi: 10.1007/s10198-019-01071-0 (PMC6544754; doi:10.1007/s10198-019-01071-0)
Supplement: Supplementary file 1 — Supplementary material 1 (PDF 140 kb) [file 10198_2019_1071_MOESM1_ESM.pdf]

Towards a Central-Eastern European EQ-5D-3L Population Norm: Comparing Data from Hungarian, Polish and Slovenian Population Studies

Zsombor Zrubka, Dominik Golicki, Valentina Prevolnik-Rupel, Petra Baji, Fanni Rencz, Valentin Brodszky, László Gulácsi, Márta Péntek

Correspondence: Zsombor Zrubka, Department of Health Economics Corvinus University of Budapest, Fővám tér 8., H-1093 Budapest, Hungary e-mail: zsombor.zrubka@uni-corvinus.hu ; phone: +36-1-482-5308

Journal: The European Journal of Health Economics

Supplementary Table S1

HUNGARY			Age group	18-24	25-34	35-44	45-54	55-64	65+	Total
Female			Sample N	207	376	315	311	182	31	1,422
	Dimension	Education	Problem level							
	Mobility	Low	Any problems	0,0%	25,0%	50,0%	62,5%	66,7%		44,4%
			Severe problems	0,0%	0,0%	0,0%	0,0%	0,0%		0,0%
		Middle	Any problems	9,6%	8,0%	16,7%	33,1%	52,4%	46,7%	23,5%
			Severe problems	0,0%	0,0%	0,0%	0,0%	0,0%	0,0%	0,0%
		High	Any problems	3,1%	7,7%	11,5%	29,4%	18,7%	43,8%	13,8%
			Severe problems	0,0%	0,0%	0,7%	0,7%	0,0%	0,0%	0,3%
	Self-care	Low	Any problems	0,0%	25,0%	25,0%	12,5%	11,1%		13,9%
			Severe problems	0,0%	0,0%	0,0%	0,0%	0,0%		0,0%
		Middle	Any problems	1,4%	0,7%	0,0%	3,8%	7,3%	0,0%	2,2%
			Severe problems	0,0%	0,0%	0,0%	0,0%	0,0%	0,0%	0,0%
		High	Any problems	0,0%	0,4%	0,7%	2,8%	0,0%	6,2%	0,9%
			Severe problems	0,0%	0,0%	0,7%	0,0%	0,0%	0,0%	0,1%
	Usual activities	Low	Any problems	0,0%	25,0%	37,5%	50,0%	44,4%		33,3%
			Severe problems	0,0%	0,0%	0,0%	0,0%	11,1%		2,8%
		Middle	Any problems	5,5%	5,1%	7,1%	18,8%	36,6%	6,7%	13,2%
			Severe problems	0,0%	0,0%	0,0%	0,6%	0,0%	0,0%	0,2%
		High	Any problems	3,1%	3,8%	7,9%	11,2%	13,2%	6,2%	7,1%
			Severe problems	0,0%	0,0%	0,7%	2,1%	0,0%	0,0%	0,5%
	Pain/discomfort	Low	Any problems	57,1%	75,0%	87,5%	87,5%	77,8%		77,8%
			Severe problems	0,0%	0,0%	12,5%	0,0%	11,1%		5,6%
		Middle	Any problems	39,7%	35,0%	38,7%	58,1%	61,0%	73,3%	46,6%
			Severe problems	1,4%	0,7%	1,2%	5,0%	8,5%	0,0%	3,0%
		High	Any problems	26,8%	25,1%	30,2%	34,3%	38,5%	62,5%	30,5%
			Severe problems	0,0%	0,0%	1,4%	2,1%	2,2%	0,0%	0,9%
	Anxiety/depression	Low	Any problems	42,9%	50,0%	75,0%	87,5%	55,6%		63,9%
			Severe problems	0,0%	0,0%	12,5%	12,5%	11,1%		8,3%
		Middle	Any problems	27,4%	46,0%	39,3%	45,0%	50,0%	33,3%	42,0%
			Severe problems	4,1%	3,6%	2,4%	7,5%	4,9%	13,3%	4,7%
		High	Any problems	22,8%	31,1%	30,9%	32,9%	24,2%	25,0%	29,0%
			Severe problems	1,6%	0,9%	2,2%	3,5%	0,0%	0,0%	1,6%

Supplementary Table S1 contd.

HUNGARY			Age group	18-24	25-34	35-44	45-54	55-64	65+	Total
Male			Sample N	90	190	183	198	155	43	859
	Dimension	Education	Problem level							
	Mobility	Low	Any problems	50,0%	0,0%	100,0%	0,0%	0,0%		40,4%
			Severe problems	0,0%	0,0%	33,3%	0,0%	0,0%		8,6%
		Middle	Any problems	0,0%	5,7%	16,0%	27,0%	32,6%	47,2%	20,5%
			Severe problems	0,0%	0,0%	1,1%	0,0%	2,3%	0,0%	0,6%
		High	Any problems	5,8%	5,0%	11,6%	23,5%	29,7%	28,1%	14,5%
			Severe problems	0,0%	0,8%	0,0%	0,0%	0,9%	0,0%	0,4%
	Self-care	Low	Any problems	50,0%	0,0%	33,3%	0,0%	0,0%		23,1%
			Severe problems	0,0%	0,0%	0,0%	0,0%	0,0%		0,0%
		Middle	Any problems	0,0%	0,0%	2,1%	3,6%	4,7%	36,6%	6,6%
			Severe problems	0,0%	0,0%	1,1%	0,0%	0,0%	0,0%	0,2%
		High	Any problems	0,0%	0,8%	0,0%	3,5%	2,7%	2,0%	1,3%
			Severe problems	0,0%	0,0%	0,0%	0,0%	0,0%	0,0%	0,0%
	Usual activities	Low	Any problems	0,0%	0,0%	66,7%	0,0%	0,0%		17,2%
			Severe problems	0,0%	0,0%	0,0%	0,0%	0,0%		0,0%
		Middle	Any problems	2,8%	2,9%	6,4%	13,5%	16,3%	41,9%	12,7%
			Severe problems	0,0%	0,0%	1,1%	0,0%	0,0%	0,0%	0,2%
		High	Any problems	5,8%	2,5%	4,7%	7,1%	13,5%	4,1%	5,4%
			Severe problems	0,0%	0,0%	0,0%	0,0%	0,9%	0,0%	0,1%
	Pain/discomfort	Low	Any problems	0,0%	100,0%	100,0%	0,0%	100,0%		48,5%
			Severe problems	0,0%	0,0%	0,0%	0,0%	0,0%		0,0%
		Middle	Any problems	27,8%	12,9%	35,1%	47,7%	58,1%	63,0%	39,7%
			Severe problems	0,0%	0,0%	0,0%	2,7%	2,3%	36,6%	5,6%
		High	Any problems	17,3%	16,0%	15,1%	37,6%	44,1%	34,2%	24,5%
			Severe problems	0,0%	0,0%	1,2%	2,4%	2,7%	2,0%	1,1%
	Anxiety/depression	Low	Any problems	0,0%	0,0%	66,7%	50,0%	0,0%		28,5%
			Severe problems	0,0%	0,0%	33,3%	0,0%	0,0%		8,6%
		Middle	Any problems	27,8%	18,6%	29,8%	31,5%	30,2%	41,9%	29,4%
			Severe problems	2,8%	1,4%	3,2%	2,7%	2,3%	0,0%	2,2%
		High	Any problems	23,1%	17,6%	22,1%	20,0%	27,9%	12,2%	20,0%
			Severe problems	1,9%	0,0%	2,3%	0,0%	4,5%	0,0%	1,2%

Supplementary Table S1 contd.

POLAND			Age group	18-24	25-34	35-44	45-54	55-64	65+	Total
Female			Sample N	218	304	348	316	416	491	2,093
	Dimensions	Education	Problem levels							
	Mobility	Low	Any problems	0,0%	40,0%	19,0%	20,7%	40,3%	62,7%	51,2%
			Severe problems	0,0%	0,0%	0,0%	0,0%	0,0%	2,4%	1,5%
		Middle	Any problems	4,8%	6,6%	6,4%	19,0%	30,9%	58,5%	23,4%
			Severe problems	0,0%	0,0%	0,0%	1,5%	0,0%	1,9%	0,6%
		High	Any problems	3,6%	2,8%	7,8%	14,9%	29,9%	52,2%	15,3%
			Severe problems	0,0%	0,0%	0,0%	1,1%	1,1%	0,0%	0,3%
	Self-care	Low	Any problems	0,0%	20,0%	9,5%	10,3%	14,9%	36,8%	28,0%
			Severe problems	0,0%	0,0%	0,0%	0,0%	0,0%	3,3%	2,1%
		Middle	Any problems	2,1%	0,8%	1,2%	6,0%	12,2%	21,2%	8,3%
			Severe problems	0,0%	0,0%	0,0%	1,5%	0,0%	1,9%	0,6%
		High	Any problems	0,0%	0,6%	1,3%	5,7%	9,2%	16,4%	4,5%
			Severe problems	0,0%	0,0%	0,0%	0,0%	1,1%	0,0%	0,2%
	Usual activities	Low	Any problems	100,0%	40,0%	9,5%	24,1%	34,3%	56,1%	46,1%
			Severe problems	0,0%	0,0%	0,0%	0,0%	0,0%	7,1%	4,5%
		Middle	Any problems	5,3%	5,7%	8,1%	18,5%	24,8%	42,0%	19,2%
			Severe problems	0,5%	0,0%	1,7%	2,0%	1,1%	4,2%	1,7%
		High	Any problems	0,0%	2,8%	5,2%	16,1%	18,4%	35,8%	11,2%
			Severe problems	0,0%	0,0%	0,0%	0,0%	2,3%	1,5%	0,5%
	Pain/discomfort	Low	Any problems	50,0%	60,0%	47,6%	58,6%	68,7%	81,1%	74,1%
			Severe problems	0,0%	0,0%	4,8%	0,0%	4,5%	11,8%	8,6%
		Middle	Any problems	19,1%	28,7%	35,3%	53,5%	59,2%	82,1%	49,1%
			Severe problems	0,5%	0,0%	0,0%	3,5%	2,3%	8,0%	2,7%
		High	Any problems	14,3%	22,0%	29,9%	40,2%	59,8%	74,6%	37,7%
			Severe problems	0,0%	0,0%	0,0%	1,1%	1,1%	4,5%	0,8%
	Anxiety/depression	Low	Any problems	100,0%	80,0%	57,1%	48,3%	52,2%	60,8%	58,3%
			Severe problems	0,0%	0,0%	4,8%	0,0%	0,0%	6,1%	4,2%
		Middle	Any problems	19,7%	19,7%	24,3%	43,0%	40,8%	52,4%	35,2%
			Severe problems	0,5%	1,6%	0,0%	2,5%	1,5%	2,8%	1,6%
		High	Any problems	17,9%	16,9%	19,5%	29,9%	40,2%	49,3%	26,5%
			Severe problems	0,0%	0,0%	0,0%	1,1%	1,1%	0,0%	0,3%

Supplementary Table S1 contd.

POLAND			Age group	18-24	25-34	35-44	45-54	55-64	65+	Total
Male			Sample N	238	309	299	294	377	327	1,844
	Dimensions	Education	Problem levels							
	Mobility	Low	Any problems	0,0%	18,2%	4,2%	15,0%	50,9%	64,9%	43,2%
			Severe problems	0,0%	0,0%	0,0%	0,0%	1,9%	3,1%	1,8%
		Middle	Any problems	3,2%	3,7%	6,6%	17,2%	33,8%	52,6%	17,9%
			Severe problems	0,0%	0,0%	0,0%	0,5%	0,4%	2,3%	0,4%
		High	Any problems	0,0%	7,4%	2,5%	7,5%	23,0%	32,1%	12,2%
			Severe problems	0,0%	0,0%	0,0%	0,0%	1,6%	0,0%	0,2%
	Self-care	Low	Any problems	0,0%	9,1%	4,2%	5,0%	26,4%	37,5%	24,0%
			Severe problems	0,0%	0,0%	0,0%	0,0%	3,8%	2,3%	1,8%
		Middle	Any problems	0,5%	0,5%	3,1%	4,1%	14,1%	22,8%	6,8%
			Severe problems	0,0%	0,0%	0,5%	0,5%	0,8%	3,4%	0,7%
		High	Any problems	0,0%	1,9%	1,3%	3,8%	6,6%	9,4%	3,8%
			Severe problems	0,0%	0,0%	0,0%	0,0%	1,6%	0,0%	0,2%
	Usual activities	Low	Any problems	25,0%	0,0%	8,3%	15,0%	45,3%	55,7%	38,3%
			Severe problems	0,0%	0,0%	0,0%	0,0%	5,7%	4,6%	3,2%
		Middle	Any problems	3,6%	4,7%	11,2%	12,7%	25,5%	40,7%	15,4%
			Severe problems	0,0%	0,0%	1,0%	0,9%	2,3%	3,9%	1,2%
		High	Any problems	0,0%	3,7%	2,5%	9,4%	13,1%	18,9%	7,8%
			Severe problems	0,0%	0,0%	0,0%	1,9%	3,3%	0,0%	0,8%
	Pain/discomfort	Low	Any problems	12,5%	9,1%	37,5%	50,0%	69,8%	86,4%	64,0%
			Severe problems	0,0%	0,0%	0,0%	5,0%	9,4%	6,3%	5,3%
		Middle	Any problems	11,7%	18,4%	28,6%	47,5%	60,8%	72,0%	38,6%
			Severe problems	0,0%	0,5%	1,0%	0,5%	3,0%	3,5%	1,3%
		High	Any problems	12,5%	13,9%	24,1%	26,4%	52,5%	63,9%	30,6%
			Severe problems	0,0%	0,9%	0,0%	1,9%	4,9%	1,6%	1,5%
	Anxiety/depression	Low	Any problems	25,0%	27,3%	16,7%	35,0%	52,8%	56,6%	45,4%
			Severe problems	0,0%	9,1%	0,0%	0,0%	3,8%	3,1%	2,9%
		Middle	Any problems	10,4%	13,7%	26,5%	32,6%	41,1%	50,3%	28,4%
			Severe problems	0,5%	0,0%	0,5%	1,8%	1,5%	2,2%	1,0%
		High	Any problems	25,0%	16,7%	15,2%	26,4%	39,3%	32,7%	23,7%
			Severe problems	12,5%	0,0%	0,0%	0,0%	3,3%	0,0%	0,8%

Supplementary Table S1 contd.

SLOVENIA			Age group	18-24	25-34	35-44	45-54	55-64	65+	Total
Female			Sample N	66	71	91	59	44	66	397
	Dimensions	Education	Problem levels							
	Mobility	Low	Any problems	0,0%	40,0%	60,0%	85,7%	63,6%	86,2%	73,0%
			Severe problems	0,0%	0,0%	0,0%	0,0%	0,0%	3,4%	1,6%
		Middle	Any problems	0,0%	10,5%	11,9%	44,1%	34,6%	63,3%	22,1%
			Severe problems	0,0%	0,0%	0,0%	0,0%	0,0%	0,0%	0,0%
		High	Any problems	12,5%	0,0%	4,5%	16,7%	28,6%	71,4%	13,3%
			Severe problems	0,0%	0,0%	0,0%	0,0%	0,0%	0,0%	0,0%
	Self-care	Low	Any problems	0,0%	20,0%	20,0%	57,1%	36,4%	48,3%	39,7%
			Severe problems	0,0%	0,0%	0,0%	0,0%	0,0%	3,4%	1,6%
		Middle	Any problems	0,0%	5,3%	10,2%	14,7%	19,2%	33,3%	11,5%
			Severe problems	0,0%	0,0%	0,0%	0,0%	0,0%	0,0%	0,0%
		High	Any problems	12,5%	0,0%	4,5%	5,6%	14,3%	28,6%	6,7%
			Severe problems	0,0%	0,0%	0,0%	0,0%	0,0%	0,0%	0,0%
	Usual activities	Low	Any problems	0,0%	40,0%	70,0%	71,4%	63,6%	79,3%	69,8%
			Severe problems	0,0%	0,0%	10,0%	14,3%	0,0%	10,3%	7,9%
		Middle	Any problems	8,8%	21,1%	20,3%	44,1%	34,6%	66,7%	28,3%
			Severe problems	0,0%	2,6%	0,0%	0,0%	3,8%	0,0%	0,8%
		High	Any problems	25,0%	7,1%	4,5%	27,8%	14,3%	57,1%	16,7%
			Severe problems	0,0%	0,0%	0,0%	0,0%	0,0%	0,0%	0,0%
	Pain/discomfort	Low	Any problems	0,0%	40,0%	70,0%	85,7%	72,7%	89,7%	77,8%
			Severe problems	0,0%	0,0%	10,0%	28,6%	9,1%	0,0%	6,3%
		Middle	Any problems	15,8%	44,7%	37,3%	61,8%	69,2%	76,7%	45,1%
			Severe problems	0,0%	2,6%	0,0%	0,0%	0,0%	0,0%	0,4%
		High	Any problems	50,0%	10,7%	36,4%	50,0%	57,1%	71,4%	36,7%
			Severe problems	0,0%	0,0%	0,0%	0,0%	0,0%	0,0%	0,0%
	Anxiety/depression	Low	Any problems	0,0%	40,0%	60,0%	85,7%	36,4%	58,6%	55,6%
			Severe problems	0,0%	0,0%	20,0%	0,0%	9,1%	0,0%	4,8%
		Middle	Any problems	17,5%	34,2%	32,2%	32,4%	57,7%	50,0%	34,0%
			Severe problems	0,0%	0,0%	0,0%	2,9%	3,8%	3,3%	1,2%
		High	Any problems	75,0%	32,1%	31,8%	27,8%	28,6%	28,6%	34,4%
			Severe problems	0,0%	0,0%	0,0%	0,0%	0,0%	0,0%	0,0%

Supplementary Table S1 contd.

SLOVENIA			Age group	18-24	25-34	35-44	45-54	55-64	65+	Total
Male			Sample N	49	65	55	55	48	39	311
	Dimensions	Education	Problem levels							
	Mobility	Low	Any problems	0,0%	33,3%	50,0%	80,0%	100,0%	70,3%	55,8%
			Severe problems	0,0%	0,0%	0,0%	0,0%	0,0%	0,0%	0,0%
		Middle	Any problems	7,1%	4,8%	28,6%	41,0%	60,0%	53,7%	31,8%
			Severe problems	0,0%	0,0%	2,9%	0,0%	0,0%	0,0%	0,6%
		High	Any problems	0,0%	17,6%	12,5%	18,2%	21,4%	60,0%	22,1%
			Severe problems	0,0%	0,0%	0,0%	0,0%	0,0%	0,0%	0,0%
	Self-care	Low	Any problems	33,3%	16,7%	25,0%	20,0%	75,0%	53,1%	36,8%
			Severe problems	0,0%	0,0%	0,0%	0,0%	0,0%	6,3%	2,1%
		Middle	Any problems	0,0%	0,0%	11,4%	17,9%	30,0%	22,6%	13,4%
			Severe problems	0,0%	0,0%	2,9%	0,0%	0,0%	0,0%	0,6%
		High	Any problems	0,0%	5,9%	0,0%	0,0%	7,1%	20,0%	4,7%
			Severe problems	0,0%	0,0%	0,0%	0,0%	0,0%	0,0%	0,0%
	Usual activities	Low	Any problems	0,0%	16,7%	50,0%	80,0%	100,0%	70,3%	52,0%
			Severe problems	0,0%	16,7%	0,0%	0,0%	50,0%	0,0%	8,2%
		Middle	Any problems	19,0%	28,6%	31,4%	38,5%	53,3%	46,3%	35,9%
			Severe problems	0,0%	0,0%	0,0%	0,0%	0,0%	0,0%	0,0%
		High	Any problems	25,0%	11,8%	6,2%	9,1%	7,1%	40,0%	12,2%
			Severe problems	0,0%	0,0%	0,0%	0,0%	0,0%	0,0%	0,0%
	Pain/discomfort	Low	Any problems	33,3%	33,3%	50,0%	80,0%	100,0%	76,6%	61,7%
			Severe problems	33,3%	16,7%	0,0%	20,0%	0,0%	0,0%	10,7%
		Middle	Any problems	33,3%	33,3%	37,1%	51,3%	70,0%	65,5%	47,6%
			Severe problems	0,0%	0,0%	2,9%	7,7%	0,0%	0,0%	1,9%
		High	Any problems	25,0%	23,5%	18,8%	27,3%	35,7%	60,0%	30,4%
			Severe problems	0,0%	5,9%	0,0%	0,0%	0,0%	0,0%	0,5%
	Anxiety/depression	Low	Any problems	66,7%	33,3%	0,0%	40,0%	100,0%	40,6%	43,3%
			Severe problems	0,0%	0,0%	0,0%	0,0%	0,0%	0,0%	0,0%
		Middle	Any problems	23,8%	38,1%	25,7%	46,2%	50,0%	46,3%	38,0%
			Severe problems	2,4%	0,0%	0,0%	7,7%	0,0%	0,0%	1,7%
		High	Any problems	25,0%	29,4%	18,8%	9,1%	21,4%	40,0%	20,8%
			Severe problems	25,0%	0,0%	0,0%	0,0%	0,0%	0,0%	0,5%
