# Supplementary material for: Towards a Central-Eastern European EQ-5D-3L population norm: comparing data from Hungarian, Polish and Slovenian population studies
Source: Eur J Health Econ. 2019 May 17;20(Suppl 1):141–54. doi: 10.1007/s10198-019-01071-0 (PMC6544754; doi:10.1007/s10198-019-01071-0)

Towards a Central-Eastern European EQ-5D-3L Population Norm: Comparing Data from Hungarian, Polish and Slovenian Population Studies

Zsombor Zrubka, Dominik Golicki, Valentina Prevolnik-Rupel, Petra Baji, Fanni Rencz, Valentin Brodszky, László Gulácsi, Márta Péntek

Correspondence: Zsombor Zrubka, Department of Health Economics Corvinus University of Budapest, Fővám tér 8., H-1093 Budapest, Hungary e-mail: zsombor.zrubka@uni-corvinus.hu ; phone: +36-1-482-5308

Journal: The European Journal of Health Economics

Supplementary Figure S2 EQ-5D-3L index scores calculated with four value-sets

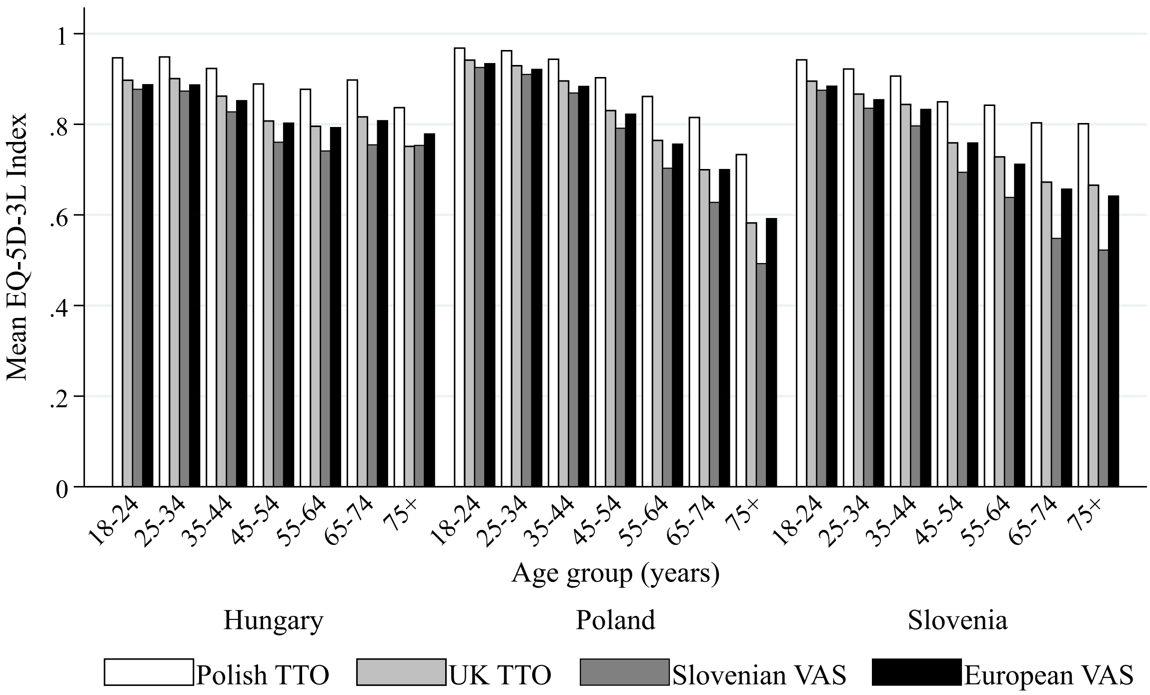

Supplement: Supplementary file 2 — Supplementary material 2 (PDF 94 kb) [file 10198_2019_1071_MOESM2_ESM.pdf]
