# Supplementary material for: Towards a Central-Eastern European EQ-5D-3L population norm: comparing data from Hungarian, Polish and Slovenian population studies
Source: Eur J Health Econ. 2019 May 17;20(Suppl 1):141–54. doi: 10.1007/s10198-019-01071-0 (PMC6544754; doi:10.1007/s10198-019-01071-0)
Supplement: Supplementary file 3 — Supplementary material 3 (PDF 93 kb) [file 10198_2019_1071_MOESM3_ESM.pdf]

Towards a Central-Eastern European EQ-5D-3L Population Norm: Comparing Data from Hungarian, Polish and Slovenian Population Studies

Zsombor Zrubka, Dominik Golicki, Valentina Prevolnik-Rupel, Petra Baji, Fanni Rencz, Valentin Brodszky, László Gulácsi, Márta Péntek

Correspondence: Zsombor Zrubka, Department of Health Economics Corvinus University of Budapest, Fővám tér 8., H-1093 Budapest, Hungary e-mail: zsombor.zrubka@uni-corvinus.hu ; phone: +36-1-482-5308

Journal: The European Journal of Health Economics

Supplementary Table S3 Effect of education, gender and age on health problems, EQ-5D-3L index scores and EQ VAS scores

			Any problems					EQ-5D-3L index scores by different value-sets				EQ VAS
			Mobility	Self-Care	Usual Activities	Pain / Discomfort	Anxiety / Depression	Polish TTO	UK TTO	Slovenian VAS	European VAS	
Education[f]	Hungary	Middle[a]	-0.221*	-0.133*	-0.180*	-0.280*	-0.195*	0.084*	0.148*	0.158*	0.137*	12.374*
		High[a]	-0.273*	-0.143*	-0.223*	-0.407*	-0.292*	0.112*	0.198*	0.212*	0.184*	17.751*
	Poland	Low[b]	0.142	0.153	0.288	-0.018	0.162	-0.014	-0.002	-0.079	-0.028	0.326
		Middle[b]	0.293	0.197	0.359*	0.206	0.257	-0.055	-0.089	-0.175	-0.110	-6.620
		High[b]	0.300	0.182	0.341*	0.263	0.304*	-0.062	-0.105	-0.190	-0.126	-8.541
	Slovenia	Low[b]	0.225	0.221	0.337	-0.069	0.004	0.041[e]	0.062	-0.062	0.008	0.000
		Middle[b]	0.167	0.156	0.288	0.065	0.095	0.065[e]	0.069[e]	-0.073[e]	0.003[e]	3.417[e]
		High[b]	0.122	0.108	0.157	0.058[e]	0.115	0.076[e]	0.078[e]	-0.051[e]	0.013[e]	2.443[e]
Gender[g]	Hungary	Female[c]	0.033*	-0.003	0.033*	0.082*	0.110*	-0.021*	-0.037*	-0.043*	-0.038*	-0.328
	Poland	Male[b]	0.011	-0.007	0.011	0.022	0.061*	-0.008	-0.015	-0.017	-0.016	0.787
		Female[b]	0.000	0.000	0.000	0.000	0.000	0.000	0.000	0.000	0.000	0.000
	Slovenia	Male[b]	0.066	-0.009	0.040	0.044	0.079	-0.025*	-0.037*	-0.043*	-0.035*	-1.731
		Female[b]	0.000	0.000	0.000	0.000	0.000	0.000	0.000	0.000	0.000	0.000
Age group[h]	Hungary	25-34 y[d]	0.027	0.003	0.004	-0.032	0.065*	-0.001	-0.002	-0.010	-0.006	0.086
		35-44 y[d]	0.098*	0.005	0.033	0.026	0.074*	-0.021*	-0.031*	-0.046*	-0.032*	-2.496*
		45-54 y[d]	0.241*	0.028*	0.097*	0.160*	0.099*	-0.056*	-0.087*	-0.114*	-0.082*	-9.374*
		55-64 y[d]	0.290*	0.028*	0.166*	0.219*	0.100*	-0.073*	-0.107*	-0.143*	-0.101*	-8.526*
		65-74 y[d]	0.318*	0.026	0.048	0.271*	0.022	-0.057*	-0.096*	-0.139*	-0.094*	-10.789*
		75+ y[d]	0.311	0.163	0.152	0.271	-0.012	-0.125	-0.173	-0.155	-0.136	-19.918*
	Poland	18-24 y[b]	-0.327	-0.191	-0.368*	-0.401*	-0.409*	0.090	0.157	0.248*	0.180	9.556
		25-34 y[b]	-0.322	-0.185	-0.354*	-0.286	-0.440*	0.077	0.135	0.229*	0.161	5.893
		35-44 y[b]	-0.388*	-0.183	-0.361*	-0.255	-0.398*	0.083	0.137	0.231*	0.156	5.809
		45-54 y[b]	-0.437*	-0.180	-0.357*	-0.236	-0.305*	0.080	0.132	0.227	0.150	6.588
		55-64 y[b]	-0.327	-0.107	-0.335*	-0.166	-0.250	0.059	0.093	0.174	0.108	-0.269
		65-74 y[b]	-0.234	-0.091	-0.160	-0.114	-0.141	0.006	0.029	0.108	0.056	-1.584
		75+ y[b]	0.000	0.000	0.000	0.000	0.000	0.000	0.000	0.000	0.000	0.000
	Slovenia	18-24 y[b]	-0.231	-0.144	-0.212	-0.170[e]	-0.160[e]	-0.048[e]	-0.035	0.108	0.027	0.334[e]
		25-34 y[b]	-0.190	-0.124	-0.129[e]	-0.056[e]	-0.117[e]	-0.070[e]	-0.068[e]	0.068[e]	-0.004[e]	-3.210[e]
		35-44 y[b]	-0.172[e]	-0.074	-0.138[e]	-0.062[e]	-0.181[e]	-0.062[e]	-0.055[e]	0.073[e]	0.007[e]	-1.400
		45-54 y[b]	-0.110[e]	-0.037	-0.045[e]	-0.018[e]	-0.128	-0.082[e]	-0.082[e]	0.040[e]	-0.015[e]	-0.466
		55-64 y[b]	-0.115[e]	0.042	-0.114[e]	0.012	-0.046	-0.102[e]	-0.082[e]	0.024[e]	-0.035[e]	-6.965
		65-74 y[b]	0.008[e]	0.085	0.197[e]	0.035	0.036	-0.065[e]	-0.124[e]	-0.044[e]	-0.074[e]	-5.089
		75+ y[b]	0.000	0.000	0.000	0.000	0.000	0.000	0.000	0.000	0.000	0.000
	Const		0.273*	0.144*	0.218*	0.580*	0.420*	0.862*	0.750*	0.722*	0.753*	70.438*
		R^2	0.209	0.157	0.180	0.171	0.072	0.169	0.190	0.243	0.211	0.249
		$F_{29.6896}$	59.819	19.462	40.488	62.943	19.590	40.537	49.125	75.394	59.825	76.035
		p=	0.000	0.000	0.000	0.000	0.000	0.000	0.000	0.000	0.000	0.000

*p<0.05 using robust standard errors, [a]base level: Hungary low education, [b]base level: Hungary [c]base level: Hungary male gender, [d]base level: Hungary 18-24 y age group, [e]p<0.05,base level: Poland

[f]Wald tests for education main effect: mobility (p<0.000), self-care(p=0.0083), usual activities (p<0.000), pain/discomfort (p<0.000), anxiety/depression (p<0.000), Polish TTO (p<0.000), UK TTO (p<0.000), European VAS (p<0.000), Slovenian VAS (p<0.000), EQ VAS (p<0.000). Education-country interaction: mobility (p=0.0013), self-care(p=0.093), usual activities (p=0.0007), pain/discomfort (p<0.0011), anxiety/depression (p<0.0654), Polish TTO (p=0.0001), UK TTO (p=0.0001), European VAS (p=0.0006), Slovenian VAS (p=0.0004), EQ VAS (p=0.0004).

[g]Wald tests for gender main effect: mobility (p<0.0375), self-care(p=0.613), usual activities (p<0.0071), pain/discomfort (p<0.000), anxiety/depression (p<0.000), Polish TTO (p=0.0001), UK TTO (p<0.000), European VAS (p<0.000), Slovenian VAS (p<0.000), EQ VAS (p=0.627). Gender-country interaction: mobility (p=0.15), self-care(p=0.75), usual activities (p<0.48), pain/discomfort (p<0.49), anxiety/depression (p<0.0243), Polish TTO (p=0.089), UK TTO (p=0.0779), European VAS (p=0.0572), Slovenian VAS (p=0.0556), EQ VAS (p=0.16).

[h] Wald test for age main effect: mobility (p<0.000), self-care(p=0.0105), usual activities (p<0.000), pain/discomfort (p<0.000), anxiety/depression (p=0.0583), Polish TTO (p<0.000), UK TTO (p<0.000), European VAS (p<0.000), Slovenian VAS (p<0.000), EQ VAS (p<0.000). Age-country interaction: mobility (p<0.000), self-care(p=0.000), usual activities (p<0.000), pain/discomfort (p<0.000), anxiety/depression (p<0.000), Polish TTO (p<0.000), UK TTO (p<0.000), European VAS (p<0.000), Slovenian VAS (p<0.000), EQ VAS (p<0.000
